# Cilostazol treatment for preventing adverse cardiovascular events in patients with type 2 diabetes and coronary atherosclerosis: Long‐term follow‐up of the ESCAPE study

**DOI:** 10.1111/1753-0407.13300

**Published:** 2022-08-05

**Authors:** Minji Sohn, Eun Ju Chun, Soo Lim

**Affiliations:** ^1^ Department of Internal Medicine Seoul National University Bundang Hospital, Seoul National University College of Medicine Seongnam Republic of Korea; ^2^ Department of Radiology Seoul National University Bundang Hospital, Seoul National University College of Medicine Seongnam Republic of Korea

**Keywords:** cardiovascular diseases, diabetes mellitus, type 2, platelet aggregation inhibitors, 关键词:2型糖尿病, 心血管疾病, 动脉粥样硬化, 血小板聚集抑制剂

## Abstract

**Background:**

Previously, in the ESCAPE study, a randomized controlled trial, we found that 12 months of cilostazol administration significantly decreased coronary artery stenosis and the noncalcified plaque component compared with aspirin. The goal of the current study was to evaluate the effect of cilostazol treatment on cardiovascular events up to 7 years after the end of the original study.

**Methods:**

After the end of the ESCAPE study with patients with type 2 diabetes mellitus (T2DM) and mild to moderate coronary artery stenosis, we decided to extend the ESCAPE study to investigate the long‐term effect of cilostazol and aspirin, named the ESCAPE‐extension study. The study participants had been investigated for cardiovascular events for up to 7 years, bringing the total follow‐up time to a median of 5.2 years (interquartile range 3.6‐6.7 years). Adverse events were also investigated.

**Results:**

Among 100 participants from the original study, 88 were included in this extension study. Cilostazol treatment reduced the incidence of cardiovascular events in the patients with T2DM when compared with aspirin for a 5.2‐year median follow‐up (hazard ratio 0.24; 95% CI, 0.07‐0.83). The cardiovascular benefit of cilostazol therapy was maintained along with age, sex, systolic blood pressure, low‐density lipoprotein cholesterol, and coronary artery calcium score. No serious adverse events in the cilostazol group were noted in the follow‐up period.

**Conclusions:**

In this ESCAPE‐extension study, cilostazol treatment proved its efficacy in reducing cardiovascular events compared with aspirin in diabetic patients with subclinical coronary artery disease, suggesting the beneficial role of cilostazol in the primary prevention of cardiovascular disease.

## Graphical abstract

**Highlights**

- In this ESCAPE‐extension study, cilostazol treatment reduced the incidence of adverse cardiovascular events in patients with type 2 diabetes when compared with aspirin for a 5.2‐year median follow‐up.
- During the follow‐up period, no adverse events were noted in the cilostazol group that exceeded those in the aspirin group.
- Cilostazol may be an effective antiplatelet therapy to lower cardiovascular risk in patients with type 2 diabetes who have well‐controlled low‐density lipoprotein‐cholesterol levels with statin treatment. A longer‐term study with a larger sample size is required to confirm this premise.

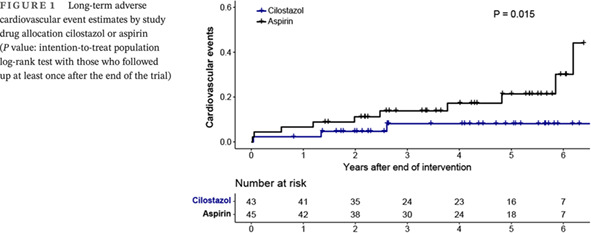

## 摘要

**背景:**

ESCAPE研究是一项随机对照试验, 在该研究中我们发现与阿司匹林相比, 西洛他唑给药12个月可显著降低冠状动脉狭窄和非钙化斑块成分。本研究旨在评估在原始研究结束7年后西洛他唑治疗对心血管事件的影响。

**方法:**

2型糖尿病合并轻中度冠状动脉狭窄患者的ESCAPE研究结束后, 我们决定延长ESCAPE研究, 以观察西洛他唑和阿司匹林的长期疗效, 命名为ESCAPE‐扩展研究。研究参与者被追踪心血管事件长达7年, 总的随访时间中位数为5.2年(四分位差范围为3.6‐6.7年)。对不良事件也进行了调查。

**结果:**

在原始研究的100名参与者中, 有88人被纳入本次扩展研究。在5.2年的中位随访中, 与阿司匹林相比, 西洛他唑治疗可降低T2DM患者的心血管事件发生率(风险比[HR] = 0.24;95%置信区间[CI] 0.07‐0.83)。西洛他唑治疗的心血管获益与年龄、性别、收缩压、低密度脂蛋白胆固醇和冠状动脉钙评分保持一致。随访期间, 西洛他唑组未发现严重不良事件。

**结论:**

在本次ESCAPE‐扩展研究中, 西洛他唑治疗糖尿病合并亚临床冠状动脉疾病患者的心血管事件较阿司匹林明显减少, 提示西洛他唑在心血管疾病一级预防中具有有益作用。

## 1 INTRODUCTION

Cardiovascular disease (CVD) is the most common complication and the leading cause of mortality in patients with type 2 diabetes mellitus (T2DM). As CVD rarely causes symptoms in the early phase, it is essential to detect its risk at that time.^1^ Therefore, management of the risk factors for atherosclerotic CVD is fundamental for managing patients with T2DM.^2^ Aspirin therapy reduces cardiovascular morbidity and death in high‐risk patients with a previous myocardial infarction or stroke^3^ and has been recommended to be prescribed for secondary prevention.^2^ However, three recent large randomized trials of aspirin therapy found no cardiovascular benefit for primary prevention and reported an increased risk of bleeding.^4^, ^5^, ^6^ Therefore, aspirin is not recommended for patients at high bleeding risk, and the role of aspirin is now limited to the secondary prevention of CVD.

Among other antiplatelet agents, cilostazol has several advantages. As a phosphodiesterase‐III inhibitor, it inhibits platelet aggregation and thrombus formation and dilates the vessels by increasing the level of cyclic adenosine monophosphate (cAMP), which plays a central role in abnormal platelet activation.^7^ A meta‐analysis has recently reported that cilostazol treatment induced beneficial effects for secondary prevention of stroke and cognitive decline.^8^ A Japanese study reported that cilostazol treatment decreased the carotid intima‐media thickness in patients with T2DM.^9^ Our group also showed that cilostazol therapy reduced the carotid plaque volume assessed by three‐dimensional ultrasonography in patients with T2DM.^10^

Previously, our group investigated the efficacy and safety of cilostazol treatment on coronary artery stenosis and plaque composition in patients with T2DM in the ESCAPE study, a randomized controlled trial.^11^ We found that 12 months of cilostazol administration significantly decreased coronary artery stenosis and the noncalcified plaque component compared with aspirin. However, because of the short‐term nature of the study, the efficacy of cilostazol on adverse cardiovascular events was not reported as a primary prevention. The goal of this study was to evaluate the effect of cilostazol treatment on such events up to 7 years after the end of the ESCAPE study.

## 2 MATERIALS AND METHODS

In the original ESCAPE study, 100 patients with T2DM and mild to moderate coronary artery stenosis, aged 35 to 78 years, and with at least one cardiovascular risk factor or suspicious symptoms were randomized to 12‐month intervention of cilostazol (200 mg/d) or aspirin (100 mg/d) between March 2014 and February 2016. More detailed information on the methods of the ESCAPE study has been reported elsewhere.^11^

Subsequently, we decided to extend the ESCAPE study to investigate the long‐term effect of cilostazol and aspirin, named the ESCAPE‐extension study. After the end of the ESCAPE study, patients had been followed up regularly in their clinics and investigated for cardiovascular events for up to 7 years, bringing the total follow‐up time to median of 5.2 years (interquartile range 3.6‐6.7).

We also collected data on subjects who underwent a cardiac multidetector computed tomography (MDCT) test at least once after the study ended. Cardiac MDCT was performed using a 256‐MDCT (Brilliance iCT; Philips Healthcare, Cleveland, Ohio) and a standard scanning protocol. The degree of coronary artery stenosis was analyzed semiautomatically comparing the contrast‐enhanced portion of the coronary lumen at the site of maximal stenosis with the mean value for the proximal and distal reference sites. The Agatston method was used to compute the coronary artery calcium score (CACS). A radiologist analyzed cardiac MDCT findings without clinical information, which means imaging analysis was conducted blind in this study. Detailed MDCT methods are specified in a previous report.^11^ Twelve participants who did not visit the hospital following the original ESCAPE study were excluded from the current analysis (Figure S1).

In the ESCAPE‐extension study, cardiovascular events were assessed as the primary endpoint. Cardiovascular events were identified as nonfatal myocardial infarction, nonfatal stroke, cardiovascular death, angina, and hospitalization for heart failure. The changes in coronary artery stenosis and CACS measured by MDCT were also compared between cilostazol and aspirin treatments. Changes in adverse cardiovascular risk factors such as glucose regulation variables, lipid profiles, and cardiac biomarkers were also investigated. Medication adherence was assessed by medication possession ratio (MPR), which was calculated as [(Sum of days' supply for medication during the period)/(Sum of days of medication required during the period)] × 100. This ESCAPE‐extension study was approved by an independent ethics committee/institutional review board (B‐2202‐744‐110).

Data are expressed as the mean and standard deviation (± SD) or N and (%). The baseline characteristics were compared using the *t* test for continuous variables or the χ^2^ test for categorical parameters. The *t* test or Wilcoxon rank sum test were used according to the distribution. Cox proportional hazards regression models with time‐dependent covariates were used to explore the adjusted risk. The original randomization was used to compare the primary outcome between cilostazol and aspirin. Further analysis using antiplatelet usage as a time‐varying value was conducted. The following covariates with known cardiovascular risk factors were tested: age; sex; status of lifestyle habits, including smoking, drinking, or exercise; systolic blood pressure (SBP), duration of diabetes, glycosylated hemoglobin, low‐density lipoprotein cholesterol (LDL‐C), high‐sensitivity C‐reactive protein (hs‐CRP), abdominal visceral fat area, coronary artery stenosis, and CACS. The final covariates were selected by backward elimination, removing factors with *P* > .1. All statistical tests were two‐sided, and statistical significance was defined as *P* < .05. Analyses were carried out with the use of R, version 4.1.0 (R Development Core Team, Vienna, Austria).

## 3 RESULTS

In this ESCAPE‐extension study, 88 subjects (43 from the cilostazol group and 45 from the aspirin group in the original ESCAPE study) were included. The mean (SD) of age was 61.0 (±9.3) years and 63% were male. Baseline characteristics are shown in Table 1. The rates of clinical characteristics, comorbidities including hypertension (~60%) and dyslipidemia (~70%), and medication usage were not significantly different between the two groups. Sodium glucose cotransporter 2 (SGLT2) inhibitors were used in five subjects in the cilostazol group alone. Glucagon‐like peptide 1 receptor agonists were not used in either group. The average MPR was similar between the two groups (94.3% ± 13.9% vs 95.1% ± 8.1%). Cilostazol medication was discontinued in one patient at the discretion of the physician.

**TABLE 1:** Characteristics of patients included in the long‐term follow‐up analysis

Variable	Cilostazol (n = 43)	Aspirin (n = 45)	*P*
Systolic blood pressure, mm Hg	126.1 (11.7)	130.0 (9.5)	.091
Diastolic blood pressure, mm Hg	73.3 (7.6)	76.3 (7.2)	.055
Body weight, kg	68.2 (11.2)	69.1 (10.3)	.709
Body mass index, kg/m^2^	25.1 (3.4)	26.0 (3.0)	.200
Abdominal subcutaneous fat area, cm^2^	153.7 (61.8)	168.1 (56.8)	.262
Abdominal visceral fat area, cm^2^	140.9 (56.6)	157.4 (52.1)	.157
Fasting glucose, mg/dL	145.3 (46.1)	143.8 (44.2)	.873
HbA1c, %	7.7 (1.4)	7.4 (1.3)	.242
Total cholesterol, mg/dL	159.7 (32.7)	171.2 (37.4)	.127
Triglycerides, mg/dL	133.4 (60.9)	154.5 (52.1)	.366
HDL‐C, mg/dL	50.0 (10.3)	50.8 (11.4)	.761
LDL‐C, mg/dL	86.8 (22.9)	95.4 (27.4)	.115
Aspartate aminotransferase, IU/L	25.5 (6.9)	27.8 (11.9)	.284
Alanine aminotransferase, IU/L	27.1 (12.6)	29.5 (14.3)	.407
Gamma‐glutamyl transferase, IU/L	32.2 (23.1)	34.5 (18.1)	.608
Creatinine, mg/dL	0.90 (0.20)	0.84 (0.22)	.179
eGFR, mL/min/1.73 m^2^	72.5 (29.1)	76.8 (30.2)	.552
hs‐CRP, mg/L	0.11 (0.12)	0.15 (0.16)	.169
CK‐MB, ng/mL	1.3 (1.0)	1.4 (1.1)	.538
Troponin‐I, ng/mL	0.05 (0.05)	0.06 (0.12)	.476
Urinary ACR, mg/g	153.4 (120.7)	285.2 (468.9)	.425
*Concurrent medication*			
ARB	28 (65.1)	21 (46.7)	.127
ACE inhibitor	2 (4.7)	4 (8.9)	.715
Statin therapy^a^	28 (65.1)	32 (71.1)	.708
High‐intensity statin	1 (2.3)	3 (6.7)	
Moderate‐intensity statin	24 (55.8)	26 (57.8)	
Low‐intensity statin	3 (7.0)	3 (6.7)	
Fibrate	0 (0.0)	4 (8.9)	.136
Metformin	38 (88.4)	38 (84.4)	.821
Insulin	12 (27.9)	12 (26.7)	.896
Sulfonylurea	32 (74.4)	28 (62.2)	.219
SGLT2 inhibitor	5 (11.6)	0 (0.0)	.058
DPP4 inhibitor	37 (86.0)	38 (84.4)	.832

*Note*: Values are presented as mean (± SD) or n (%).

Abbreviations: ACE, angiotensin‐converting enzyme; ACR, albumin to creatinine ratio; ARB, angiotensin II receptor blocker; CK‐MB, creatine kinase‐muscle/brain; DPP‐IV, dipeptidyl peptidase IV; eGFR, estimated glomerular filtration rate; HbA1c, glycosylated hemoglobin; HDL‐C, high‐density lipoprotein cholesterol; hs‐CRP, high‐sensitivity C‐reactive protein; LDL‐C, low‐density lipoprotein cholesterol; SGLT2, sodium glucose cotransporter 2.

^a^ High‐intensity statin: rosuvastatin 20 mg; moderate‐intensity statin: atorvastatin 10‐20 mg, rosuvastatin 5‐10 mg, simvastatin 20‐40 mg, pravastatin 40 mg, pitavastatin 4 mg; low‐intensity statin: simvastatin 10 mg.

**FIGURE 1:**
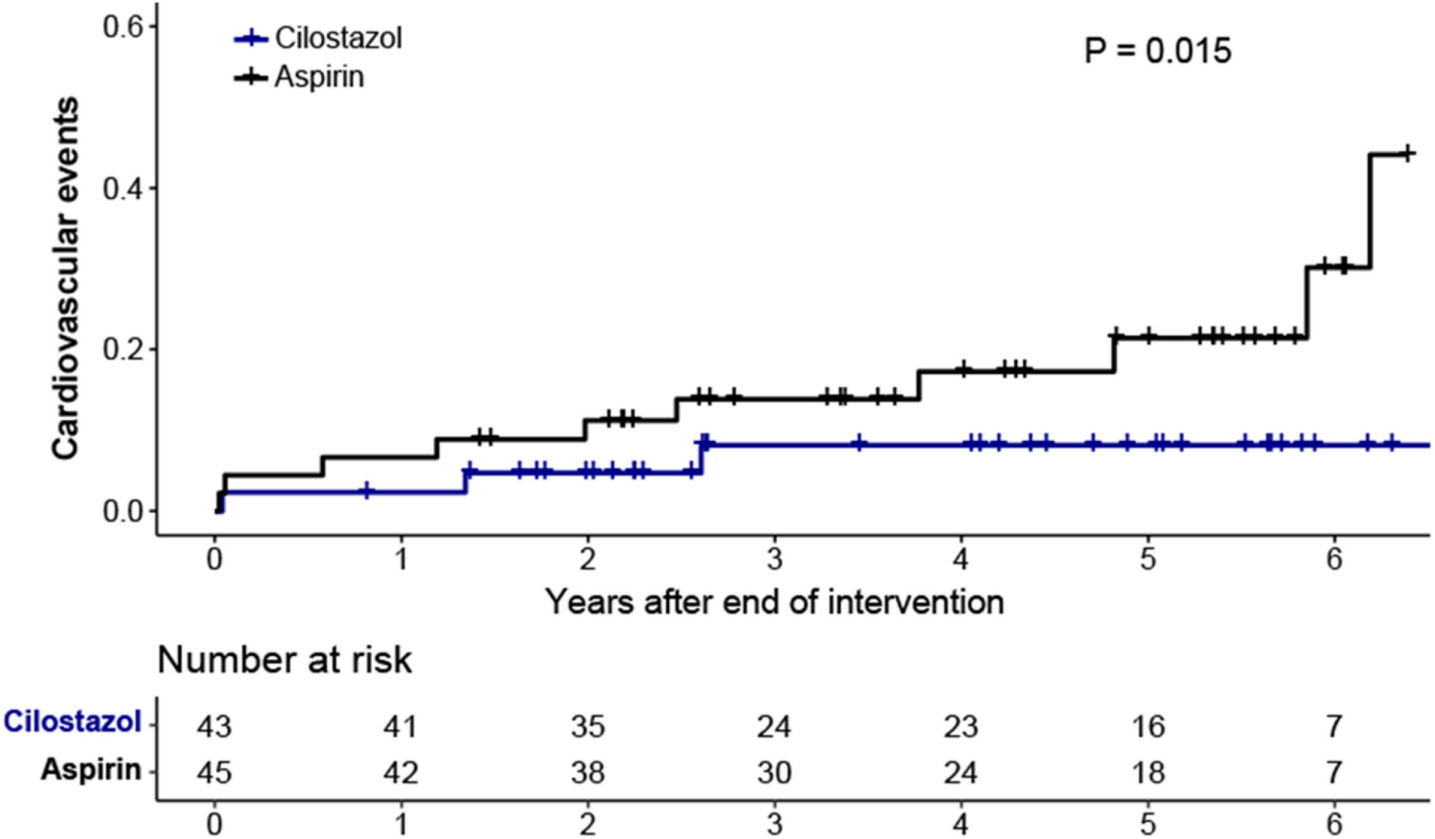
Long‐term adverse cardiovascular event estimates by study drug allocation cilostazol or aspirin (*P* value: intention‐to‐treat population log‐rank test with those who followed up at least once after the end of the trial)

During a median 5.2 years of follow‐up, the number of adverse cardiovascular events was significantly different between the two groups: 3 patients in the cilostazol group vs 12 in the aspirin group (hazard ratio [HR] 0.24; 95% CI, 0.07‐0.83) (Figure 1). In multivariable analysis, the beneficial effect of cilostazol therapy on cardiovascular events was maintained with HR 0.22 (95% CI, 0.05‐0.92) along with age, male sex, SBP, LDL‐C, and CACS (Table 2). After the positive results with cilostazol in the original ESCAPE study, four subjects switched from aspirin to cilostazol. To reflect this change, further analysis was performed using antiplatelet usage as a time‐varying value, which showed similar results (HR 0.29; 95% CI, 0.09‐0.90). Duration of diabetes, lifestyle habits, and hs‐CRP were not related to cardiovascular events (Table S1). In a further analysis using antidiabetic medication as a covariate in the regression model, the use of SGLT2 inhibitors did not affect the results.

**TABLE 2:** Independent predictors of adverse cardiovascular events with long‐term follow‐up (median 5 years)

	Nonadjusted			Fully adjusted^a^		
	HR	95% CI	*P*	HR	95% CI	*P*
Cilostazol vs aspirin	0.24	0.07‐0.83	.026	0.22	0.05‐0.92	.038
Age, y	1.08	1.02‐1.14	.007	1.11	1.04‐1.19	.003
Male	0.70	0.25‐1.96	.499	4.18	1.19‐14.68	.026
Systolic blood pressure,^b^ mm Hg	1.05	1.01‐1.09	.018	1.10	1.03‐1.17	.005
Abdominal visceral fat area,^b^ mm^2^	1.00	0.99‐1.01	.600	0.99	0.97‐1.00	.098
LDL‐C,^b^ mg/dL	1.02	0.99‐1.04	.138	1.02	1.00‐1.05	.046
Coronary artery calcium score^b^ ^,^ ^c^	1.53	0.96‐2.45	.072	2.23	1.09‐4.59	.029

Abbreviations: HR, hazard ratio; LDL‐C, low‐density lipoprotein cholesterol.

^a^ The stepwise backward elimination method with removing variables with *P* > .1 was adopted.

^b^ Time‐dependent variables were used in the Cox regression model.

^c^ HR was calculated with log‐transformed values.

**FIGURE 2:**
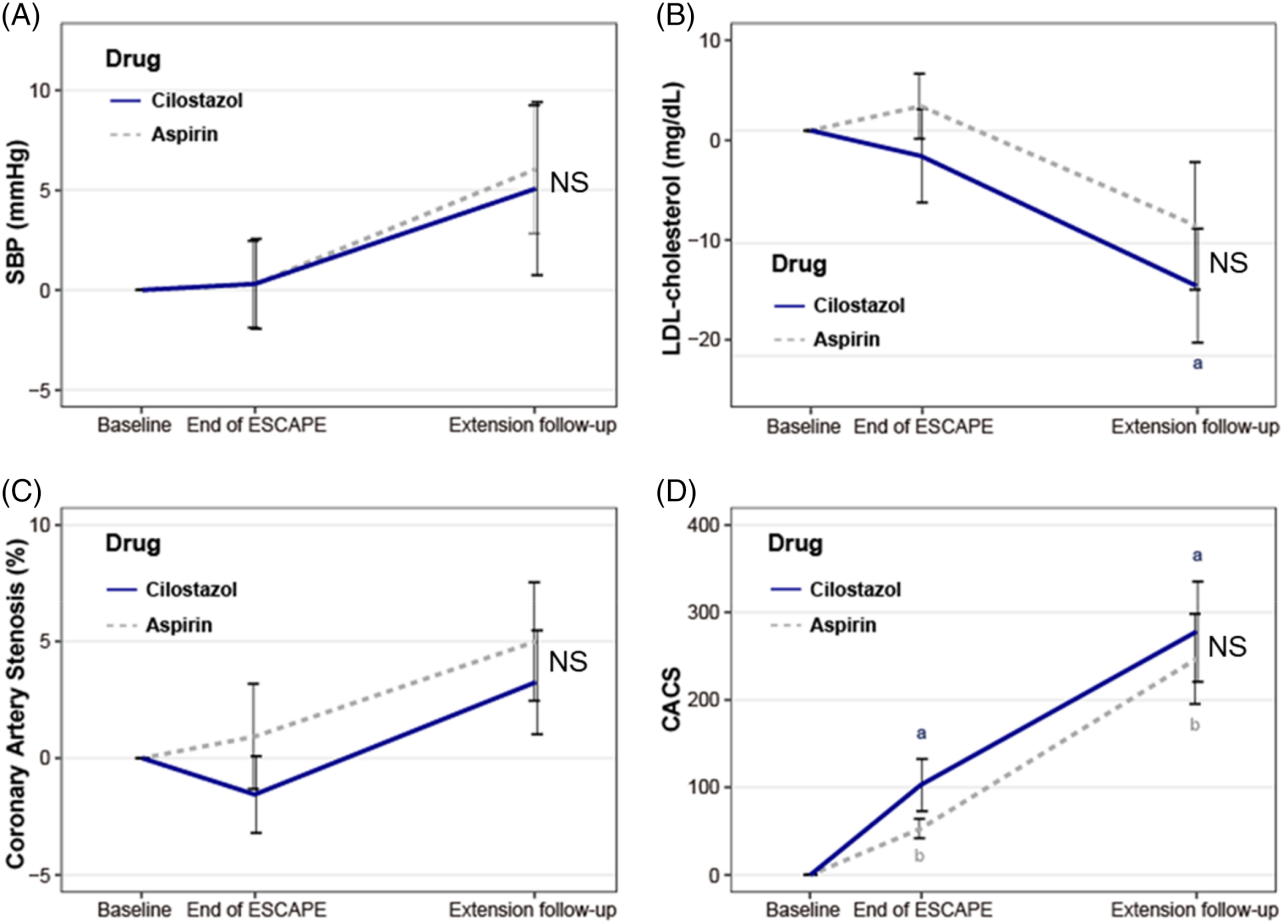
Changes in (A) systolic blood pressure (SBP), (B) low density lipoprotein (LDL)‐cholesterol, (C) coronary artery stenosis, and (D) coronary artery calcium score (CACS). Data are expressed as the mean ± SEM. *P* < .05 by paired Student's *t* test between the values at the baseline and at the observed time‐point, end of ESCAPE trial or last observation with the date of adverse cardiovascular events: a indicates baseline in the aspirin group, b indicates baseline in the cilostazol group. NS, not significant: the values did not differ between the two groups

In the assessment of cardiac MDCT and cardiovascular risk factors at the date of cardiovascular events or last observation, we found that coronary artery stenosis and CACS progressed naturally in both groups over time without statistical significance between the groups (Figure 2). Of note, SBP increased, and LDL‐C levels decreased in the ESCAPE‐extension study compared with the corresponding values assessed at the end of the original ESCAPE study.

Reported adverse events except for CVD are presented in Table 3. During the extension study period, 21 participants (48.8%) in the cilostazol group and 27 (60.0%) in the aspirin group reported adverse events, which were all mild to moderate such as dizziness and gastrointestinal discomfort. There were no cases where cilostazol medication was stopped due to intolerance, such as severe headache. One case of bleeding from the gastrointestinal tract was observed in the aspirin group. One death was confirmed in the cilostazol group due to a malignant neoplasm of the gallbladder.

**TABLE 3:** Adverse events observed in the ESCAPE‐extension study

Category	Cilostazol (n = 43)	Aspirin (n = 45)
Total subjects	21 (48.8)	27 (60.0)
Total events	27	35
Bleeding	0/27 (0.0)	1/35 (2.9)
Eye disorders	4/27 (14.8)	2/35 (5.7)
Gastrointestinal disorders	3/27 (11.1)	3/35 (8.6)
Genitourinary disorders	3/27 (11.1)	4/35 (11.4)
Hepatobiliary disorders	3/27 (11.1)	3/35 (8.6)
Mental disorders	0/27 (0.0)	2/35 (5.7)
Musculoskeletal disorders	6/27 (22.2)	8/35 (22.9)
Neoplasms benign, malignant, and unspecified	2/27 (7.4)	8/35 (22.9)
Neurological disorders	3/27 (11.1)	3/35 (8.6)
Respiratory disorders	2/27 (7.4)	0/35 (0.0)
Skin and subcutaneous tissue disorders	1/27 (3.7)	1/35 (2.9)

*Note*: Values are presented as n (%).

## 4 DISCUSSION

In this ESCAPE‐extension study, we found that cilostazol treatment reduced the incidence of adverse cardiovascular events in the patients with T2DM when compared with aspirin for a 5.2‐year median follow‐up. No adverse events in the cilostazol group were noted in the follow‐up period.

Cilostazol selectively inhibits phosphodiesterase‐IIIA, which is found in vascular smooth muscle cells and platelets. The antiplatelet actions of cilostazol are enhanced by inhibiting adenosine (A) uptake, which consequently stimulates A_2_ receptors and increases cAMP.^7^ Unlike aspirin, cilostazol can inhibit shear stress‐induced platelet aggregation.^7^ Of note, in an animal experiment, administration of cilostazol increased nitric oxide levels and improved endothelial dysfunction.^12^ Cilostazol also has favorable effects on phosphodiesterase‐V activity.^7^ The inhibition of phosphodiesterase‐V also has a protective effect against myocardial ischemic injury by increasing nitric oxide synthase activity and activating protein kinase G.^13^

Cilostazol has several additional effects that can be related to reducing the incidence of adverse cardiovascular events. It decreases triglyceride levels by 10% by activating lipoprotein lipase activity.^14^ Several studies have reported that cilostazol has anti‐inflammatory and antioxidative effects in human studies.^10^, ^11^ If a future large‐scale randomized trial supports these findings, cilostazol might be an effective option for the primary prevention of CVD in patients with T2DM and mild to moderate coronary artery stenosis.

Here, high SBP, high LDL‐C levels, and a greater burden of coronary calcium were the significant risk factors for adverse cardiovascular events. A high burden of calcification in coronary vessels is a well‐known risk for the progression of atherosclerosis and coronary heart diseases.^15^ In the present study, the CACS increased by about 10% per year, with an evident increase in young subjects and in men for a total of >350, whereas the figure was lower in women, as reported elsewhere.^16^ Notably, there was no significant difference in the progression of CACS in this study between the cilostazol and aspirin groups. This could indicate that cilostazol treatment may have a beneficial role to play in the noncalcified regions of plaque in the coronary arteries rather than in the calcified portion, as found in the original ESCAPE study.^10^

At the last observed time point in this study, there was no difference in the degree of coronary artery stenosis between the cilostazol and aspirin groups (Figure 2). This might be because the cardiac MDCT scan was not done at a time when cardiovascular events occurred, possibly decreasing the difference in the reported degree of coronary artery stenosis between the two groups.

According to recent large studies, aspirin has failed to prove its primary cardiovascular preventive role but has an increased bleeding risk compared with placebo treatment, overweighing its beneficial effects.^6^ The increased risk was substantial, particularly in elderly people. Recent guidelines suggest that for primary prevention, the use of aspirin needs to be carefully considered and might generally not be recommended.^2^ However, aspirin is still widely used in real‐world clinical practice. From this perspective, considering that LDL‐C levels were well controlled under 100 mg/dL during the observation, our study suggests that cilostazol can be considered an effective candidate to reduce cardiovascular risk in addition to statin therapy in patients with diabetes.

There are several limitations to the current study. The sample size may not be sufficient to conclude that cilostazol therapy has a positive role in primary prevention of cardiovascular events. Thus, a further large‐scale randomized trial using cilostazol is needed for this purpose. Because the follow‐up cardiac MDCT scan was done at the discretion of the physician without regular intervals, the observation period between baseline and follow‐up varied, which might affect the changes in cardiac MDCT findings.

In conclusion, previous studies showed that cilostazol treatment was effective in reducing secondary adverse events in patients who had myocardial infarction or stroke.^8^, ^17^ We reported previously that cilostazol treatment was effective in mitigating the progression of atherosclerosis in carotid and coronary arteries.^10^, ^11^ Moreover, our ESCAPE‐extension study showed that cilostazol treatment decreased the incidence of adverse cardiovascular events compared with aspirin in Korean patients with T2DM and subclinical coronary artery disease, suggesting the beneficial role of cilostazol in the primary prevention of CVD. Further large‐scale trials comparing placebo or other antiplatelet agents are warranted to evaluate the effects of cilostazol on primary prevention.

## AUTHOR CONTRIBUTIONS

S.L. conceived the study, extracted and analyzed data, and wrote the manuscript. M.S. helped conceive the study, extracted and analyzed data, and wrote the manuscript. E.J.C. analyzed the image data. M.S., E.J.C., and S.L. critically reviewed the manuscript and approved its submission. S.L. is the guarantor of this work and, as such, had full access to all the data in the study and takes responsibility for the integrity of the data and the accuracy of the data analysis.

## CONFLICT OF INTEREST

The authors have no conflicting interests relevant to this article to disclose.

## Supporting information


**Figure S1** Flow chart of the participants included in the current study.
**Table S1.** Independent predictors of adverse cardiovascular events with long‐term follow‐up (median 5 years)

## Data Availability

Data are available from the authors upon reasonable request.
