# Supplementary material for: Cilostazol treatment for preventing adverse cardiovascular events in patients with type 2 diabetes and coronary atherosclerosis: Long‐term follow‐up of the ESCAPE study
Source: J Diabetes. 2022 Aug 5;14(8):524–31. doi: 10.1111/1753-0407.13300 (PMC9426278; doi:10.1111/1753-0407.13300)
Supplement: Supplementary file 1 — Figure S1 Flow chart of the participants included in the current study. Table S1. Independent predictors of adverse cardiovascular events with long‐term follow‐up (median 5 years) [file JDB-14-524-s001.docx]

**Supplementary figure S1.** Flow chart of the participants included in the current study.

**
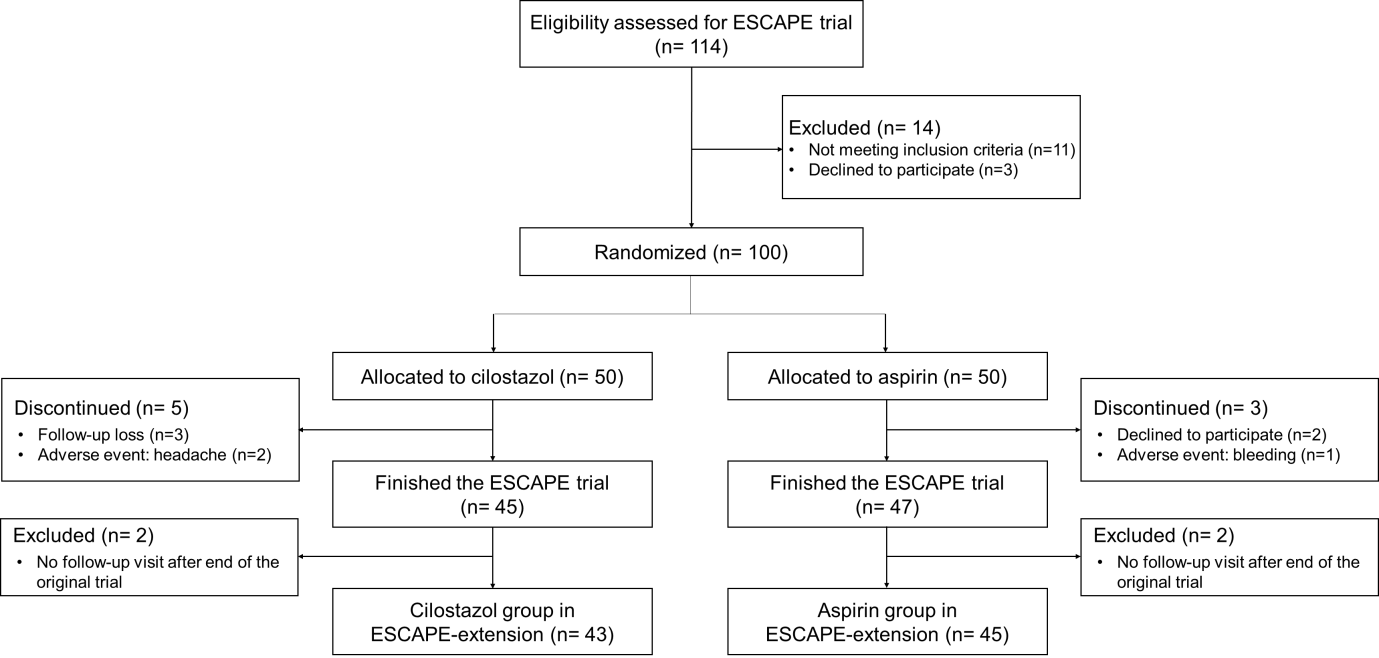
**

| **Supplementary table S1.** Independent predictors of adverse cardiovascular events with long-term follow-up (median 5 years) | | | |
| --- | --- | --- | --- |
| **Variables** | **Non-adjusted** | | |
|  | **HR** | **95% CI** | ***P*** |
| Cilostazol vs. Aspirin^a^ | 0.29 | 0.09–0.90 | 0.032 |
| Age, year | 1.08 | 1.02–1.14 | 0.007 |
| Male | 0.70 | 0.25–1.96 | 0.499 |
| Systolic blood pressure^a^, mmHg | 1.05 | 1.01–1.09 | 0.018 |
| Visceral fat area^a^, mm^2^ | 1.00 | 0.99–1.01 | 0.600 |
| LDL-cholesterol^a^, mg/dL | 1.02 | 0.99–1.04 | 0.138 |
| Coronary artery calcium score^a,b^ | 1.53 | 0.96–2.45 | 0.072 |
| Current smoker | 0.90 | 0.30–2.68 | 0.848 |
| Current drinker | 0.72 | 0.26–2.02 | 0.534 |
| Regular exercise | 0.42 | 0.14–1.24 | 0.115 |
| Diabetes duration, year | 1.02 | 0.99–1.06 | 0.225 |
| HbA1c^a^, % | 1.02 | 0.73–1.44 | 0.899 |
| hsCRP^a^, mg/dL | 0.67 | 0.06–7.06 | 0.742 |
| Coronary artery stenosis^a^, % | 1.02 | 0.99–1.06 | 0.233 |
| ^a^ Time-dependent variable was used in each Cox regression model.  ^b^ The hazard ratio (HR) was calculated with log-transformed values.  Abbreviations: LDL, low-density lipoprotein; HbA1c, glycosylated hemoglobin; hsCRP, high-sensitivity C-reactive protein | | | |
